# *Col5a3* Likely Promotes Adipogenesis of 3T3-L1 Through Oxidative Phosphorylation

**DOI:** 10.3390/genes16020165

**Published:** 2025-01-27

**Authors:** Sheng Wen, Ruimin Ren, Hanhao Yuan, Ning Gao, Jun He, Yuebo Zhang

**Affiliations:** 1 College of Animal Science and Technology, Hunan Agricultural University, Changsha 410128, China; 15885463786@stu.hunau.edu.cn (S.W.); ruimin.ren@hunau.edu.cn (R.R.); 15211029428@163.com (H.Y.); gaon@hunau.edu.cn (N.G.); 2 Key Laboratory of Livestock and Poultry Resources (Pig) Evaluation and Utilization, Ministry of Agriculture and Rural Affairs, Changsha 410128, China

**Keywords:** *Col5a3*, proliferation, adipogenesis, 3T3-L1, RNA-seq

## Abstract

Background: A recent study reported that a deficiency of *Col5a3* reduces dermal fat. However, the regulatory mechanism of the *Col5a3* gene on adipose deposition remains unclear. Methods: In this study, we assessed the effects of *Col5a3* interference on the proliferation and differentiation of 3T3-L1 preadipocytes through CCK-8, EdU staining, cell cycle detection, RT-qPCR, Western blot, a triglyceride assay, and Oil Red O staining. RNA-seq was then performed on differentiated adipocytes to identify key differentially expressed genes (DEGs) and signaling pathways. Results: *Col5a3* interference significantly promoted the proliferation of 3T3-L1 cells but inhibited their differentiation. RNA-seq analysis identified 368 DEGs, with the most significant enrichment observed in the oxidative phosphorylation pathway. Conclusions: This study demonstrates the regulatory role of *Col5a3* in the proliferation and differentiation of preadipocytes, identifying various genes regulated by *Col5a3* in adipogenesis. We speculate that *Col5a3* may influence adipogenesis through the oxidative phosphorylation pathway in 3T3-L1 cells. The findings help gain a better understanding of the molecular mechanisms underlying fat deposition and obesity-related metabolic diseases.

## 1. Introduction

Fat storage is an essential mechanism for reserving energy in humans and animals, and it is strongly correlated with sustained metabolic health [1]. Adipose tissues and adipocytes have emerged as major research areas over the past decade, both domestically and internationally. Moreover, the effective regulation of fat accumulation in pigs provides significant advantages to both farmers and consumers [2]. Vertebrate collagens, a large family of extracellular proteins, are crucial to the formation and function of virtually every organ system [3]. They play diverse roles in cell adhesion, differentiation, cell migration, as well as tissue regeneration [4]. Collagen V [col(V)] is a low-abundance fibrillar collagen, widely distributed throughout vertebrate tissues as α1(V)_2_α2(V) heterotrimers [5]. Theα1(V)α2(V)α3(V) heterotrimer has been identified in the placenta, uterus, skin, and synovial membranes; developing joint ligaments; peripheral nerves; and in the vicinity of developing skeletal muscle. Previous studies reported that the Pro-α3(V) chain is expressed at lower levels than other fibrillar collagen genes in a mouse wound healing model [6,7]. Zhang et al. [8] found that a panel of collagen members was downregulated in aged osteoblasts, including *Col12a1*, *Col5a1*, *Col5a3*, *Col8a1*, and *Col8a2*. Our previous research revealed that differential editing sites (DESites) located within the *Col5a3* gene are candidate sites highly associated with fat deposition [9]. Adipose tissue is composed of adipocyte. The proliferation and differentiation of adipocytes are also key ways for fat deposition. Previous studies found that *Col5a3* is a gene expressed in WAT, and female *Col5a3*^−/−^ mice showed a significant reduction in subcutaneous abdominal fat compared to WT female mice [10,11,12]. *Col5a3* was recognized as a hub extracellular matrix (ECM)-related gene, and the elevation of *Col5a3* is linked to the development and metastasis of bladder cancer (BLCA) [13]. Fang discovered that the *Col5a3* gene is essential for notochord and mesenchyme cell growth in the eyes and lens during zebrafish embryonic development [14]. After treatment with butyrate, the *Col5a3* gene is significantly upregulated in bovine epithelial cells [15]. The *Col5a3* gene plays an important role in stabilizing tendons [16]. *Col5a3* negatively regulates the differentiation of mouse osteoblasts [17]. Therefore, we speculate that *Col5a3* may play a key role in adipocyte differentiation and proliferation. In this study, we investigated the functional role of *Col5a3* in proliferation and adipogenesis of 3T3-L1 preadipocytes, and further explored its targeted pathway using RNA-seq. By investigating the role of *Col5a3* in adipocytes, we may gain a better understanding of the molecular mechanisms underlying fat deposition and obesity-related metabolic diseases.

## 2. Materials and Methods

### 2.1. Cell Culture

3T3-L1 cells were purchased from Aniphe (Nanjing, China) and cultured in growth medium at 37 °C with 5% CO_2_ [18]. Upon reaching 90% confluence, the cells were initiated to undergo differentiation using (DMEM with 10% FBS, 0.5 mmol/L 3-Isobuty-1-methyxanthine (IBMX) (Merck, Shanghai, China), 10 μg/mL insulin (Beyotime Biotechnology, Shanghai, China), and 1 μmol/mL dexamethasone (Merck, Shanghai, China) for 2 days. Following this, the cells were maintained in DMEM with 10% FBS and 10 μg/mL insulin for an additional 2 days. The maintenance medium was replaced every 2 days. The complete adipogenic differentiation process lasted 8 days.

### 2.2. Transfection with siRNA

The small interfering RNA (siRNA) of *Col5a3* was synthesized by GenePharma (Suzhou, China) and contained three siRNAs and a negative control (NC). Transfection was carried out with Lipofectamine 2000 Reagent (ThermoFisher, Waltham, MA, USA) when cells reached proper confluence for proliferation or differentiation. The final concentration of siRNA or negative control was 50 nM. Cells were changed into fresh growth medium 24 h post-transfection.

### 2.3. RNA Isolation and Quantitative Real-Time PCR

Total RNA was extracted using Trizol (TaKaRa, Beijing, China) and then reversed. Quantitative real-time PCR (RT-PCR) was carried out using an SYBR Green master mix and specific primers

### 2.4. Western Blot Detection

The proteins were extracted with Cell Lysis buffer (Tris-HCI) obtained from Bioss (Beijing, China). Bicinchoninic Acid (BCA) assay was used to determined protein concentrations. Following this, 10 ug of protein underwent electrophoresis on SDS–polyacrylamide gels (YEASEN, Shanghai, China). Subsequently, the separated proteins were transferred onto a polyvinylidene fluoride (PVDF) membrane (Merck, Shanghai, China). The PVDF membrane was then blocked for 2 h, followed by overnight incubation with primary antibodies at 4 °C. The PVDF membrane was then washed and exposed to a secondary antibody for 2 h. Visualization of the protein bands was achieved using chemiluminescence reagents from Meilunbio (Dalian, China) and quantification was carried out with ImageJ2 (ver. 1.54f) software. Antibodies for CCNE1, CCND1, FABP4, and COL5A3 were obtained from Aifang (Changsha, China). The β-actin antibody was sourced from Cell Signaling Technology (Danvers, MA, USA). PPARγ and Lipoprotein lipase (LPL) antibodies were procured from Bioss (Beijing, China). The CDK4 antibody was acquired from Huabio (Hangzhou, China).

### 2.5. Flow Cytometry

3T3-L1 cells were transfected with *Col5a3* siRNA using Lipofectamine 2000 once they reached 60–70% confluence. After 24 h, cells were harvested and processed according to the cell cycle and apoptosis detection kit (Meilunbio, Dalian, China). Cell cycle analysis was performed using flow cytometry (BD FACSCelesta, BD Biosciences, Franklin Lakes, NJ, USA).

### 2.6. Oil Red O Staining

Mature adipocytes were stained using Oil Red O (Merck, Shanghai, China) after a series of PBS washes. After three PBS washes, the cells were fixed in 4% paraformaldehyde for 10 min, followed by re-fixation with fresh paraformaldehyde for 1 h, and again washed three times with PBS. The cells were then exposed to 2 mL of 60% isopropanol for 2–5 min, air-dried on a sterile surface, and then stained with 1% filtered Oil Red O solution in the dark for 10 min. After staining, the cells were washed several times with PBS until the solution was clear. Lipid droplets in the adipocyte cytoplasm were observed under an inverted fluorescence microscope (Carl Zeiss AG, Jena, Germany).

### 2.7. EdU Assay

3T3-L1 cells were plated at a concentration of 5000 cells per well in a 96-well plate. When the cells reached 50% confluence, they were transfected with siRNA. After 36 h, cell proliferation was assessed using the EdU staining kit (BioScience, Shanghai, China). The images were captured with an inverted fluorescence microscope (Carl Zeiss AG, Jena, Germany) and analyzed and quantified using ImageJ2.

### 2.8. Cell Counting Kit-8 Assay

Cell proliferation was assessed using the Cell Counting Kit-8 (Meilunbio, Dalian, China). 3T3-L1 cells were plated at 5000 cells per well in a 96-well plate. Once reaching approximately 40% confluence, the cells were transiently transfected with *Col5a3*-targeting vectors for knockdown. Viability was monitored at 0, 24, and 48 h post-transfection using the CCK-8 kit at 37 °C for 4 h. The optical density (OD) at 450 nm was measured using a microplate reader (Infinite M200PRO TECAN, Teacan, Männedorf, Switzerland).

### 2.9. Triglyceride Determination

To analyze triglyceride levels, 3T3-L1 cells were trypsinized, neutralized with an equal volume of complete culture medium. After centrifugation at 800 rpm for 5 min, the cell pellets were gathered. Subsequently, 200 μL of cell lysis buffer was introduced, followed by a 10 min incubation period. The supernatant obtained after centrifugation was used for triglyceride determination following a 10 min heating step at 70 °C to establish a standard curve. Triglyceride levels were quantified by measuring the absorbance at 550 nm using a multifunctional microplate reader (Infinite M200PRO TECAN, Männedorf, Switzerland).

### 2.10. RNA Sequencing and Data Processing

Six libraries, representing treatment and NC groups, were constructed. RNA was extracted using Trizol reagent (TaKara, Dalian, China), and its quality was assessed with a NANODROP 200 (Thermo Fisher Scientific, Grand Island, NE, USA). After passing quality checks, cDNA libraries were prepared and sequenced on a NovaSeqX Plus sequencer (Illumina, Sangon Biotech, Shanghai, China). DESeq2 were used to identified DEGs (|log2FC| ≥ 1, padj < 0.05). The GO and KEGG enrichment analyses for DEGs were performed using a website “https://www.bioinformatics.com.cn/ (accessed on 23 November 2024)”. Terms and pathways with *p*-values ≤ 0.05 were considered statistically significant.

### 2.11. Statistical Analysis

The data are presented as mean ± SD. Differences between groups were assessed using the Student’s *t*-test, with significance defined as *p* < 0.05. “*n* = 3” represents three technical replicates.

## 3. Results

### 3.1. Efficiency of Col5a3 Interference

**Figure 1:**
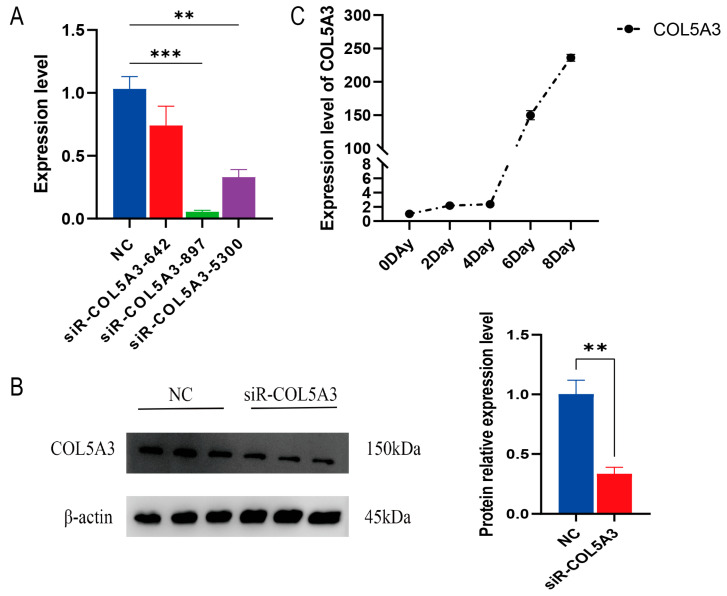
Assessment of *Col5a3* knockdown effectiveness: The efficiency of *Col5a3* knockdown was evaluated at the mRNA level (**A**) and protein level (**B**). (**C**) Expression levels of *Col5a3* during adipocyte differentiation. Data are presented as mean ± SD (*n* = 3). Significance levels: ** *p* < 0.01, *** *p* < 0.005.

To study the effect of *Col5a3* on 3T3-L1 preadipocyte, we separately transfected three interference fragments targeting *Col5a3* into cells. After 36 h of culture, cells were collected and assessed interference efficiency. In the treatment with si-*Col5a3*-897, mRNA levels decreased by over 90% compared to the NC, demonstrating the highest interference efficiency (Figure 1A), while protein levels decreased by more than 50% (Figure 1B). Therefore, si-*Col5a3*-897 was used in subsequent experiments. Furthermore, we observed a significant increase in the expression levels of *Col5a3* with differentiation time (Figure 1C).

### 3.2. Col5a3 Blunts Cell Proliferation

**Figure 2:**
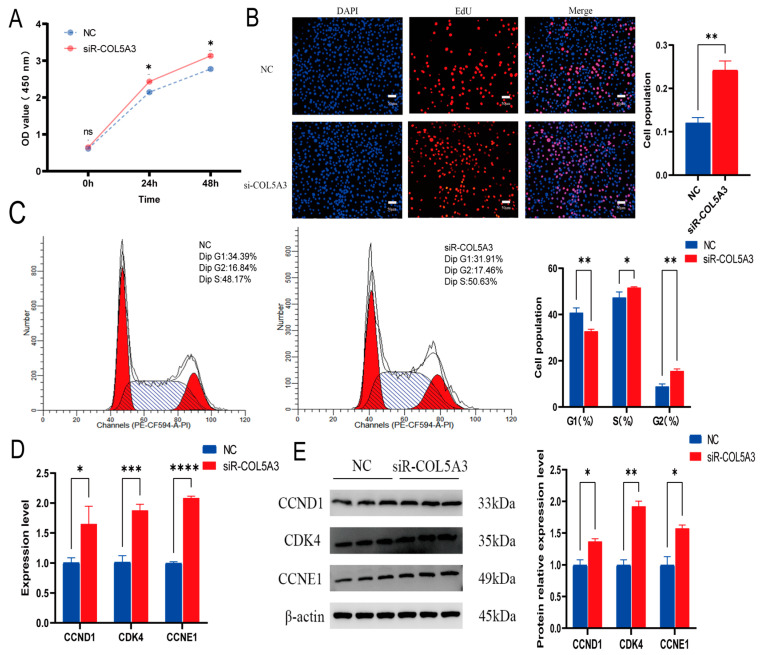
Disruption of *Col5a3* enhances 3T3-L1 preadipocyte proliferation. The following assays were conducted: (**A**) CCK-8 cell viability assay measuring absorbance at 450 nm. (**B**) EdU assay for proliferation activity. (**C**) Flow cytometry analysis for cell cycle detection. (**D**) Evaluation of relative mRNA expression levels of *CCND1*, *CDK4*, and *CCNE1* genes. (**E**) Protein expression of CCND1, CDK4, and CCNE1 genes using β-actin as the house-keeping protein. Results are displayed as mean ± SD (*n* = 3). Statistical significance: ns *p* > 0.05, * *p* < 0.05, ** *p* < 0.01, *** *p* < 0.005, **** *p* < 0.001.

We first tested the effect of *Col5a3* in cell proliferation. 3T3-L1 cells were transfected with si-*Col5a3*-897 and viability was monitored at 0, 24, and 48 h. Results showed that cell viability was significantly increased compared to NC (Figure 2A), suggesting that *Col5a3* inhibits cell proliferation. Moreover, the EdU results also confirmed that depletion of *Col5a3* has a positive effect on cell proliferation (Figure 2B). Flow cytometry revealed a significant decrease in the number of cells in the G1 phase, along with a significant increase in the S and G2 phase (Figure 2C). We also detected that the cell proliferation marker genes *CCND1*, *CDK4*, and *CCNE1* were significantly upregulated in si-*Col5a3* (Figure 2D). The Western blot showed a similar trend (Figure 2E). These results indicate that *Col5a3* promotes cell proliferation.

### 3.3. Col5a3 Promotes Adipogenesis

**Figure 3:**
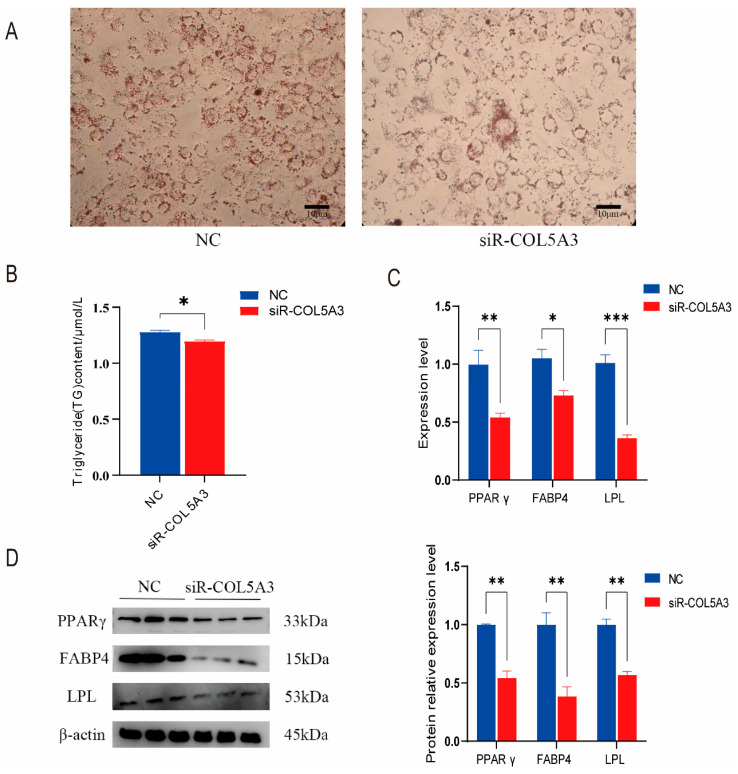
Downregulation of *Col5a3* hinders the differentiation of 3T3-L1 preadipocytes. Experiments conducted: (**A**) Oil Red O staining on the 8th day of 3T3-L1 preadipocyte differentiation. (**B**) Assessment of triglyceride content. (**C**) Analysis of the relative mRNA expression of *PPARγ*, *FABP4*, and *LPL* genes. (**D**) Protein expression analysis of PPARγ, FABP4, and LPL genes with β-actin as the internal control. Results are presented as mean ± SD (*n* = 3). Significance levels: * *p* < 0.05, ** *p* < 0.01, *** *p* < 0.005.

To investigate the impact of *Col5a3* on adipogenic differentiation in 3T3-L1 preadipocytes, *Col5a3* was knocked down for 24 h, followed by differentiation for 8 days. Cells were then stained with Oil Red O and assessed for triglyceride content (Figure 3A,B). Results showed that *Col5a3* knockdown inhibited differentiation and triglyceride accumulation. RT-qPCR analysis revealed a significant decrease in expression of *PPARγ*, *FABP4*, and *LPL* (Figure 3C). Western blot also showed a significant decreased in those proteins (Figure 3D). These results suggest that *Col5a3* promotes the adipogenesis of 3T3-L1 preadipocytes. To investigate the effect of *Col5a3* on adipogenic differentiation of 3T3-L1 preadipocytes, *Col5a3* was knocked down for 24 h, followed by differentiation for 8 days. Cells were then stained with Oil Red O and assessed for triglyceride content (Figure 3A,B). Results showed that *Col5a3* knockdown inhibited adipogenic differentiation and triglyceride accumulation. RT-qPCR analysis revealed a significant decrease in the expression of PPARγ, FABP4, and LPL.

### 3.4. Summary of RNA-Seq Data and Identification of Differentially Expressed Genes (DEGs)

**Figure 4:**
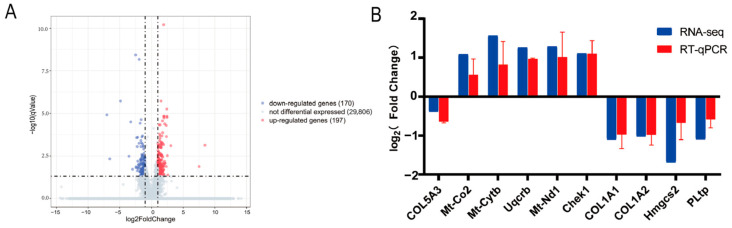
Detection of RNA-seq data and differentially expressed genes (DEGs): (**A**) Visualization of differentially expressed genes through a volcano plot. (**B**) Confirmation of DEG expression levels via RT-qPCR post siRNA transfection for six days, with *n* = 3 replicates. The NC group is used as the reference, and log2 (fold change) is calculated as the ratio of gene expression in the siR group to that in the NC group.

To evaluate the effect of *Col5a3* on the transcriptomic profile during differentiation, and the TPM method was employed for gene expression quantification. After quality control and filtering, 46.95 G clean reads were obtained, with Q20 content exceeding 97%. Over 90% of the reads aligned to the reference genome (Table S1), indicating high-quality sequencing data suitable for further analysis. Differential expression analysis identified 368 DEGs between the NC group and siR group, comprising 170 downregulated and 198 upregulated genes (Figure 4A). To validate the accuracy of the RNA-seq data, we randomly selected nine differentially expressed genes (DEGs) and assessed their expression levels using RT-qPCR. We confirmed the expression levels of these genes in the NC group and siR group. Figure 4B demonstrates that the expression patterns of the nine DEGs were in agreement with the RNA-seq data, thereby confirming the reliability of the RNA-seq results. 

### 3.5. Regulation of Adipogenic Differentiation by Col5a3, Probably Through the Oxidative Phosphorylation Pathway

**Figure 5:**
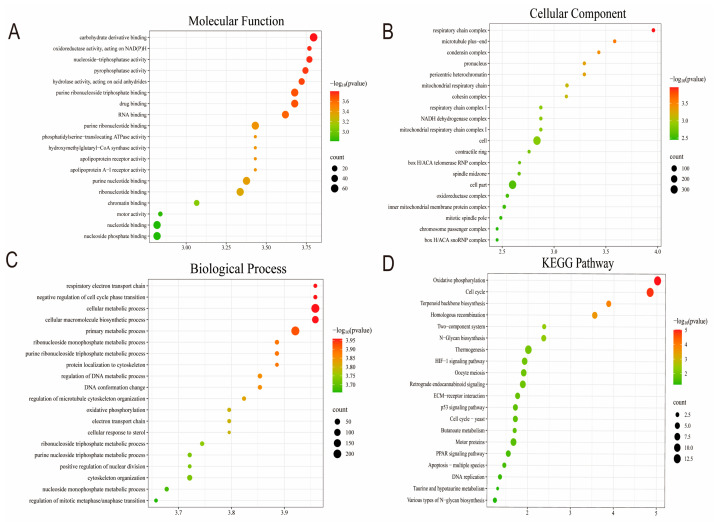
GO and KEGG pathway analysis for differentially expressed genes (DEGs): (**A**) Molecular functions. (**B**) Cellular components. (**C**) Biological processes. (**D**) Enrichment analysis of DEGs in KEGG pathways.

**Figure 6:**
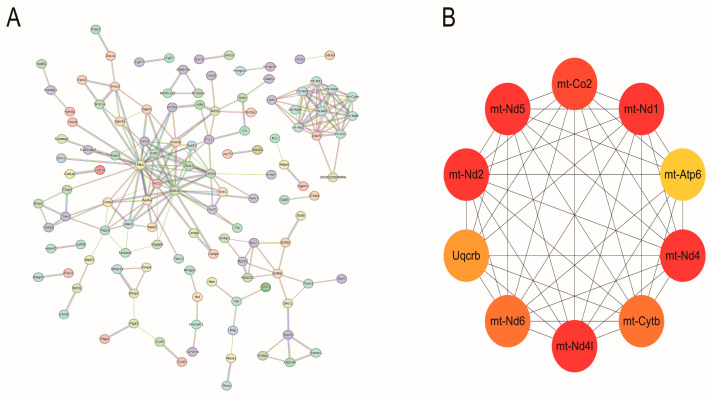
PPI network and hub gene screening: (**A**) PPI network construction. (**B**) cytoHubba-selected 10 key genes.

In order to investigate the functional of the DEGs, Gene Ontology (GO) enrichment analysis on the 367 DEGs (Table S2) was performed. These DEGs were classified into three categories, including 850 terms for Molecular Function (MF), 613 terms for Cellular Component (CC), and 4251 terms for Biological Process (BP). Figure 5A–C displays the top 20 GO terms (*p* < 0.05). The molecular functions of the genes were primarily involved in “carbohydrate derivative binding”, “oxidoreductase activity, acting on NAD(P)H”, and “nucleoside-triphosphatase activity”. Cellular components related to these genes include “respiratory chain complex”, “condensin complex”, and “pericentric heterochromatin”. Biological processes include “respiratory electron transport chain”, “purine ribonucleoside triphosphate metabolic process “, and “oxidative phosphorylation”. In the KEGG pathway analysis, oxidative phosphorylation was also enriched by DEGs and had the lowest *p*-value. Moreover, the DEGs were significantly enriched in the pathways participating in the regulation of preadipocyte differentiation, including the thermogenesis and PPAR signaling pathways (Figure 5D, Table S3). Furthermore, enrichment analysis was performed on the downregulated and upregulated DEGs resulting from *Col5a3* knockdown, separately. The downregulated DEGs were enriched in some KEGG pathways such as Extracellular structures and Carbohydrate transport and metabolism, while the upregulated DEGs were enriched in multiple KEGG pathways including Cell cycle control, cell division, chromosome partitioning, and Cytoskeleton. The 367 differentially expressed genes selected were analyzed in the String database to obtain protein–protein interaction results. The Network Analyzer tool in Cytoscape was used to calculate undirected scores for various nodes in the protein interaction network, and the protein interaction network was visualized for analysis based on the obtained degree values. Furthermore, using the Cytoscape plugin cytoHubba and selecting the MCC algorithm, 10 key genes, *mt-Co2*, *mt-Nd4l*, *mt-Nd1*, *mt-Cytb*, *mt-Nd6*, *mt-Atp6*, *Uqcrb*, *mt-Nd4*, *mt-Nd2*, and *mt-Nd5* were identified in crucial positions within the PPI protein interaction network (Figure 6A,B). These genes represent crucial constituents of the oxidative phosphorylation pathway. These results suggest that *Col5a3* may exert corresponding biological functions by interacting with the expression products of the aforementioned genes.

## 4. Discussion

Adipogenesis is a complex biochemical process involving the differentiation of preadipocytes, followed by their proliferation and maturation into adipocytes. These preadipocytes, originating from the existing adipocyte population, undergo development in response to specific stimuli. Therefore, understanding the molecular mechanisms driving adipogenesis is essential for gaining insights into this process. The distribution of preadipocytes in the different phases of the cell cycle is an indicator of their proliferation rate. An increase in the l number of the S phase indicates enhanced cell proliferation activity [18]. Huang et al. found that downregulation of *Col5a3* gene expression significantly reduces the proliferative potential of tumor cells [19]. In our study, on the hand, transfection with siRNA remarkably increases the number of cells in the S phase, proving that *Col5a3* inhibited the cell cycle. This result indicates that the role of *Col5a3* in cell proliferation varies among different cell types. On the other hand, the EdU and CCK-8 assays showed that *Col5a3* also downregulated the proliferation of preadipocytes. The impact of *Col5a3* on preadipocyte expansion was further demonstrated by the expression of cell proliferation markers. Knockdown of *Col5a3* significantly increased the mRNA and protein levels of proliferation-related genes, such as *CCND1* and *CDK4*. *CCND1* is a member of the highly conserved cyclin family that regulates *CDK* kinases [20].

As is well known, adipocyte differentiation involves the sequential regulation of multiple transcription factors, with *PPARγ* playing a central role in this process [21]. *LPL* and *FABP4*, as downstream targets of *PPARγ*, are directly or indirectly regulated by *PPARγ* to sustain lipogenesis [22]. These genes were commonly considered as adipocytes differentiation markers. We found that knockdown of *Col5a3* not only inhibited the mRNA levels of *PPARγ*, *FABP4*, and *LPL*, but also the protein expression. Additionally, knockdown of *Col5a3* decreased the number and aggregation of lipid droplets, which impaired adipocyte differentiation. Our results are consistent with those of Huang et al. [9], demonstrating the reliability of our findings.

RNA-seq was used to identify the downstream genes and key signaling pathways of *Col5a3*. Analysis of data from transfected siRNA and NC cells revealed significant enrichment. GO analysis showed notable enrichment in biological processes associated with adipocyte energy metabolism, including “respiratory electron transport chain”, “purine ribonucleoside triphosphate metabolic process”, and “oxidative phosphorylation”. KEGG pathway analysis identified the regulation of oxidative phosphorylation, thermogenesis, and the PPAR signaling pathway by the DEGs. These results suggest that *Col5a3* plays a role in adipogenesis by modulating DEGs and their respective KEGG pathways in 3T3-L1 cells. A protein–protein interaction (PPI) network of the DEGs was constructed. Ten hub genes, including *mt-Co2, mt-Nd4l, mt-Nd1, mt-Cytb, mt-Nd6, mt-Atp6, Uqcrb, mt-Nd4, mt-Nd2, and mt-Nd5*, were identified as significant regulators involved in differentiation of adipocytes, potentially contributing to the disparities in differentiation of adipocytes between the NC group and siR group. The PPAR signaling pathway plays an important role in adipose tissue function and energy metabolism through regulation of the genes expressed in lipid metabolism [19,23]. The PPAR signaling pathway is essential for lipid metabolism, as it regulates lipid synthesis, degradation, and transport [24,25,26]. All these pathways have a proven role in adipogeneses. The PPAR signaling pathway is essential for lipid metabolism, as it regulates lipid synthesis, degradation, and transport [27,28]. Previous studies have demonstrated that oxidative phosphorylation is vital in the accumulation of intramuscular fat within muscle tissue [14]. Chronic high-fructose/high-fat diet consumption reduces the hepatic mitochondrial oxidative phosphorylation pathway in mice [29]. Kanano et al. [30] found that soy isoflavones reduce lipid deposition in muscle cells by enhancing the expression of oxidative phosphorylation-related genes such as *mt-Cycs*, *mt-Cytb*, and *ATP5B* in *C2C12* myotubes. Furthermore, Silvie Timmers and Xiu-Fang Chen et al. demonstrated that blocking oxidative phosphorylation induces muscle fat accumulation in rats [31]. These results indicate that oxidative phosphorylation exerts a negative regulatory effect on fat formation. In this study, significant enrichment of the oxidative phosphorylation pathway was observed in the knockdown of *Col5a3* compared to the NC group, consistent with previous research findings. This presents new evidence that the oxidative phosphorylation and PPAR signaling pathways perform crucial roles in adipogenesis. In conclusion, we speculate that *Col5a3* may regulate the differentiation of 3T3-L1 cells by downregulating the oxidative phosphorylation pathway.

## 5. Conclusions

In conclusion, this study demonstrates the regulatory function of the *Col5a3* gene in preadipocyte proliferation and differentiation. We found that knockdown of *Col5a3* could promote the proliferation and depress the differentiation of 3T3-L1 preadipocytes. Furthermore, we identified various genes regulated by *Col5a3* in adipogenesis and found that *Col5a3* may impact the adipogenesis of 3T3-L1 cells through the oxidative phosphorylation pathway. These results present novel avenues for modulating fat deposition and contribute to a deeper understanding of the molecular mechanisms in adipogenesis.

## Data Availability

The raw sequence data reported in this paper have been deposited in the Genome Sequence Archive (Genomics, Proteomics & Bioinformatics 2021), National Genomics Data Center (Nucleic Acids Res 2022), and China National Center for Bioinformation/Beijing Institute of Genomics, Chinese Academy of Sciences (GSA: CRA020715) and are publicly accessible at “https://ngdc.cncb.ac.cn/gsa (accessed on 25 November 2024)”.

## Supplementary Materials

The supporting information can be accessed from https://www.mdpi.com/article/10.3390/genes16020165/s1; Table S1: Summary of sequencing reads aligned with the Mus musculus genome; Table S2: GO analysis of DEGs; Table S3: KEGG pathway analysis of DEGs; Figure S1: Bioinformatics analysis of downregulated genes. (A) Enriched molecular functions terms for DEGs. (B) Enriched cellular components terms for DEGs. (C) Enriched biological processes terms for DEGs. (D) KEGG enrichment analysis of DEGs; Figure S2: Bioinformatics analysis of upregulated genes. (A) Enriched molecular functions terms for DEGs. (B) Enriched cellular components terms for DEGs. (C) Enriched biological processes terms for DEGs. (D) KEGG enrichment analysis of DEGs.

## References

[1] Zha A., Li W., Wang J., Bai P., Qi M., Liao P., Tan B.E., Yin Y. (2024). Trimethylamine oxide supplementation differentially regulates fat deposition in liver, longissimus dorsi muscle and adipose tissue of growing-finishing pigs. Anim. Nutr..

[2] Jiang J., Zhou J., Chen J., Wei X., Lu T., Chi H., Zhao R. (2007). Effect of chicken egg yolk antibody against adipose tissue plasma membranes on carcass composition and lipogenic hormones and enzymes in pigs. Livest. Sci..

[3] Vuorio E., de Crombrugghe B. (1990). The family of collagen genes. Annu. Rev. Biochem..

[4] Fang M., Adams J.S., Mcmahan B.L., Brown R.J., Oxford J.T. (2010). The expression patterns of minor fibrillar collagens during development in zebrafish. Gene Expr. Patterns.

[5] Fichard A., Kleman J., Ruggiero F. (1995). Another look at collagen V and XI molecules. Matrix Biol..

[6] Chernousov M.A., Stahl R.C., Carey D.J. (1996). Schwann cells secrete a novel collagen-like adhesive protein that binds N-syndecan. J. Biol. Chem..

[7] Sumiyoshi H., Kitamura H., Matsuo N., Tatsukawa S., Ishikawa K., Okamoto O., Fujikura Y., Fujiwara S., Yoshioka H. (2012). Transient Expression of Mouse Pro-α3(V) Collagen Gene (Col5a3) in Wound Healing. Connect. Tissue Res..

[8] Zhang X., Zhao G., Zhang Y., Wang J., Wang Y., Cheng L., Sun M., Rui Y. (2018). Activation of JNK signaling in osteoblasts is inversely correlated with collagen synthesis in age-related osteoporosis. Biochem. Biophys. Res. Commun..

[9] Zhang Y., Liu X., Zhang L., Wang L., He J., Ma H., Wang L. (2022). Preliminary identification and analysis of differential RNA editing between higher and lower backfat thickness pigs using DNA-seq and RNA-seq data. Anim. Genet..

[10] Huang G., Ge G., Wang D., Gopalakrishnan B., Butz D.H., Colman R.J., Nagy A., Greenspan D.S. (2011). α3(V) collagen is critical for glucose homeostasis in mice due to effects in pancreatic islets and peripheral tissues. J. Clin. Investig..

[11] Nguyen D.V., Nguyen O.C., Malau-Aduli A. (2021). Main regulatory factors of marbling level in beef cattle. Vet. Anim. Sci..

[12] Cheng Z., Li X., Bao S., Yamada T., Cao G., Liu J., Chen A., Tong B. (2023). Effects of the CDC10 (Septin 7) Gene on the Proliferation and Differentiation of Bovine Intramuscular Preadipocyte and 3T3-L1 Cells. Animals.

[13] Zhao H., Chen Z., Fang Y., Su M., Xu Y., Wang Z., Gyamfi M.A., Zhao J. (2022). Prediction of Prognosis and Recurrence of Bladder Cancer by ECM-Related Genes. J. Immunol. Res..

[14] Chen X.F., Wang L., Wu Y.Z., Song S.Y., Min H.Y., Yang Y., He X., Liang Q., Yi L., Wang Y. (2018). Effect of puerarin in promoting fatty acid oxidation by increasing mitochondrial oxidative capacity and biogenesis in skeletal muscle in diabetic rats. Nutr. Diabetes.

[15] Chun T.H., Hotary K.B., Sabeh F., Saltiel A.R., Allen E.D., Weiss S.J. (2006). A pericellular collagenase directs the 3-dimensional development of white adipose tissue. Cell.

[16] Xiao J., Bai X., Liao L., Zhou M., Peng J., Xiang Q., Ren Z., Wen H., Jiang Z., Tang Z. (2019). Hydrogen sulfide inhibits PCSK9 expression through the PI3K/Akt-SREBP-2 signaling pathway to influence lipid metabolism in HepG2 cells. Int. J. Mol. Med..

[17] Sun H., Liu X., Long S.R., Wang T., Ge H., Wang Y., Yu S., Xue Y., Zhang Y., Li X. (2019). Antidiabetic effects of pterostilbene through PI3K/Akt signal pathway in high fat diet and STZ-induced diabetic rats. Eur. J. Pharmacol..

[18] Yang M., Jiang J., Ren R., Gao N., He J., Zhang Y. (2024). Role of ADAR1 on Proliferation and Differentiation in Porcine Preadipocytes. Animals.

[19] Auwerx J. (1999). PPARγ, the ultimate thrifty gene. Diabetologia.

[20] Huang G., Ge G., Izzi V., Greenspan D.S. (2017). α3 Chains of type V collagen regulate breast tumour growth via glypican-1. Nat. Commun..

[21] Consortium T.U. (2018). UniProt: A worldwide hub of protein knowledge. Nucleic Acids Res..

[22] Wang X., Liang C., Li A., Cheng G., Long F., Khan R., Wang J., Zhang Y., Wu S., Wang Y. (2022). RNA-Seq and lipidomics reveal different adipogenic processes between bovine perirenal and intramuscular adipocytes. Adipocyte.

[23] Guo H., Khan R., Abbas Raza S.H., Suhail S.M., Khan H., Khan S.B., Abd El-Aziz A.H., Zan L. (2021). RNA-Seq Reveals Function of Bta-miR-149-5p in the Regulation of Bovine Adipocyte Differentiation. Animals.

[24] Gu H., Zhou Y., Yang J., Li J., Peng Y., Zhang X., Miao Y., Jiang W., Bu G., Hou L. (2021). Targeted overexpression of PPARγ in skeletal muscle by random insertion and CRISPR/Cas9 transgenic pig cloning enhances oxidative fiber formation and intramuscular fat deposition. FASEB J..

[25] Lodhi I.J., Semenkovich C.F. (2014). Peroxisomes: A nexus for lipid metabolism and cellular signaling. Cell Metab..

[26] Li Z., Hassan M.Q., Jafferji M., Aqeilan R.I., Garzon R., Croce C.M., van Wijnen A.J., Stein J.L., Stein G.S., Lian J.B. (2009). Biological functions of miR-29b contribute to positive regulation of osteoblast differentiation. J. Biol. Chem..

[27] Jacobson K.R., Lipp S., Acuna A., Leng Y., Bu Y., Calve S. (2020). Comparative Analysis of the Extracellular Matrix Proteome across the Myotendinous Junction. J. Proteome Res..

[28] Wilson D.F. (2017). Oxidative phosphorylation: Regulation and role in cellular and tissue metabolism. J. Physiol..

[29] Chiang Morales M.D., Chang C.Y., Le V.L., Huang I.T., Tsai I.L., Shih H.J., Huang C.J. (2022). High-Fructose/High-Fat Diet Downregulates the Hepatic Mitochondrial Oxidative Phosphorylation Pathway in Mice Compared with High-Fat Diet alone. Cells.

[30] Kitamura K., Erlangga J.S., Tsukamoto S., Sakamoto Y., Mabashi-Asazuma H., Iida K. (2020). Daidzein promotes the expression of oxidative phosphorylation- and fatty acid oxidation-related genes via an estrogen-related receptor α pathway to decrease lipid accumulation in muscle cells. J. Nutr. Biochem..

[31] Timmers S., Nabben M., Bosma M., van Bree B., Lenaers E., van Beurden D., Schaart G., Westerterp-Plantenga M.S., Langhans W., Hesselink M.K.C. (2012). Augmenting muscle diacylglycerol and triacylglycerol content by blocking fatty acid oxidation does not impede insulin sensitivity. Proc. Natl. Acad. Sci. USA.

