# Supplementary figures and images for: Col5a3 Likely Promotes Adipogenesis of 3T3-L1 Through Oxidative Phosphorylation
Source: Genes (Basel). 2025 Jan 27;16(2):165. doi: 10.3390/genes16020165 (PMC11855316; doi:10.3390/genes16020165)

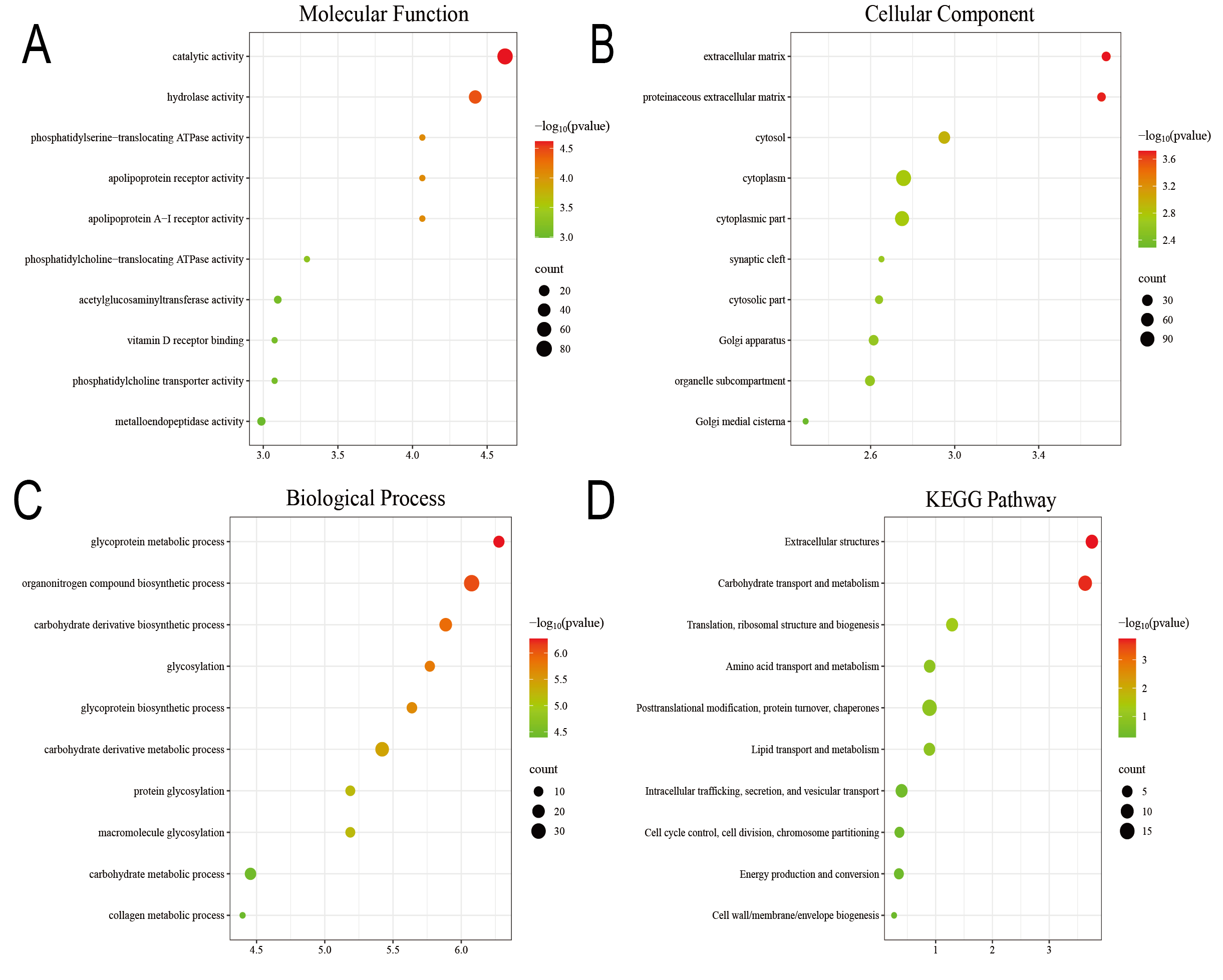

Supplement: Supplementary file 1 [file genes-16-00165-s001.zip › Figure S1.Bioinformatics analysis of genes that are differentially expressed and downregulated.png]

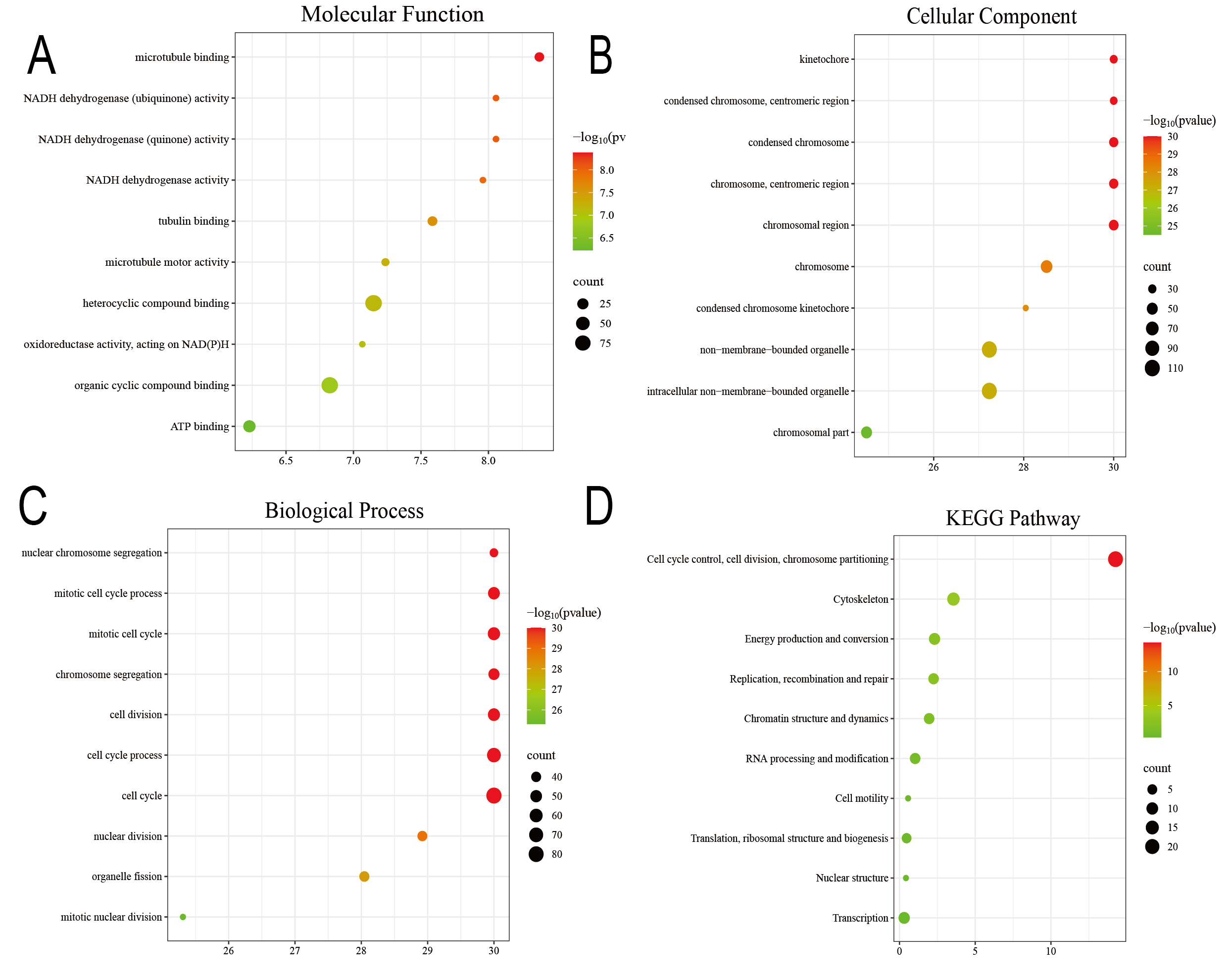

Supplement: Supplementary file 1 [file genes-16-00165-s001.zip › Figure S2.Bioinformatics analysis of genes that are differentially expressed and upregulated.png]
